# Sexual Orientation Discrimination in Early Adolescents

**DOI:** 10.1001/jamanetworkopen.2024.37985

**Published:** 2024-10-07

**Authors:** Jason M. Nagata, Jennifer H. Wong, Christiane K. Helmer, Sydnie K. Domingue, Joan E. Shim, Abubakr Al-Shoaibi

**Affiliations:** 1 Department of Pediatrics, University of California, San Francisco

## Abstract

This cross-sectional study examined the prevalence of sexual orientation discrimination and its sociodemographic correlates among a large, diverse sample of early adolescents in the US.

## Introduction

Sexual minority adolescents are more likely to experience sexual orientation discrimination, possibly leading to isolation, depressive symptoms, self-harm, and suicidal ideation.^1^ Bullying may contribute to sexual orientation discrimination by perpetuating stereotypes and reinforcing negative attitudes toward sexual minorities. In 2021, 35% of gay or lesbian and 36% of bisexual high school students experienced bullying compared with 18% of their heterosexual counterparts.^2^ However, little is known about sexual orientation discrimination in early adolescence, when sexual identity often begins to develop. Our study aimed to examine the prevalence of sexual orientation discrimination and sociodemographic factors associated with discrimination among a large, diverse sample of early adolescents in the US.

## Methods

Cross-sectional data from the Adolescent Brain Cognitive Development (ABCD) Study (year 2, 2018-2020, adolescents aged 10-13 years) were analyzed.^3^ Additional information on the ABCD Study’s design and measures is available in the eMethods in Supplement 1. Institutional review board approval was obtained from each study site and the University of California, San Diego. Written informed parental or caregiver consent was provided. This cross-sectional study complied with the checklist for the CROSS guideline. Sexual orientation discrimination was measured from the Perceived Discrimination Scale,^4,5^ which measures adolescents’ perception of being treated unfairly because of sociodemographic factors. All participants were asked, “In the past 12 months, have you felt discriminated against because someone thought you were gay, lesbian, or bisexual?” Responses included “yes” and “no.”

A multivariable Poisson regression analysis was conducted to estimate the associations of age, sex, sexual orientation, race and ethnicity (Asian, Black, Latino or Hispanic, Native American, White, and other), religiosity, household income, and highest parental education level with sexual orientation discrimination. The aforementioned variables were parent and/or participant reported. Analyses were adjusted for the independent variables and study site. Stata, version 18.0 (StataCorp LLC) was used to conduct our analyses.

## Results

Our sample consisted of 9345 early adolescents, with 4.9% of the total sample experiencing sexual orientation discrimination. The prevalence of sexual orientation discrimination varied by sexual orientation (Figure) and was highest among adolescents who identified as gay or bisexual (37.4%). In Poisson regression analyses of the total sample, male sex was associated with a higher prevalence of experiencing sexual orientation discrimination compared with female sex (adjusted prevalence ratio [aPR], 1.25; 95% CI, 1.03-1.52) (Table). Those who identified as gay or bisexual (aPR, 13.97; 95% CI, 11.32-17.23), maybe gay or bisexual (aPR, 8.08; 95% CI, 6.23-10.46), refused to answer (aPR, 4.00; 95% CI, 2.11-7.58), or did not understand the question (aPR, 1.81; 95% CI, 1.02-3.21) had a higher prevalence of experiencing sexual orientation discrimination compared with those who identified as heterosexual. Lower household income was associated with a higher prevalence of experiencing sexual orientation discrimination compared with households earning $200 000 or greater. Lower parental education was associated with a higher prevalence of experiencing sexual orientation discrimination compared with higher parental education (aPR, 1.45; 95% CI, 1.09-1.93). In regression analyses limited to sexual minority adolescents, findings were mostly similar.

**Figure.**
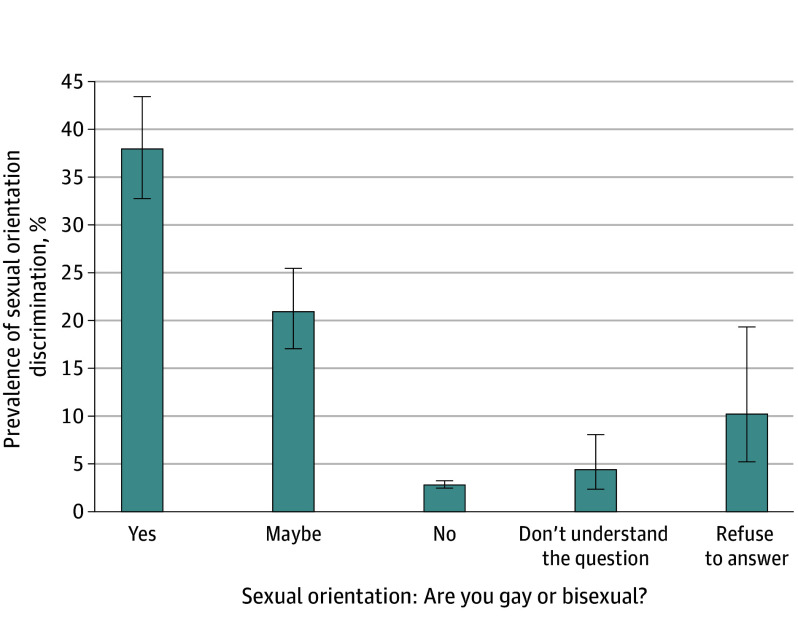
Distribution of Sexual Orientation Discrimination by Sexual Orientation

**Table.** Sociodemographic Characteristics and Associations With Experiencing Sexual Orientation Discrimination in the Adolescent Brain Cognitive Development Study (N = 9345)^a^

Characteristic	No. (%)	No./total No. (%)^b^	aPR (95% CI)		
			Total	Among gay or bisexual and maybe gay or bisexual
Age, mean (SD), y	12.0 (0.7)		1.08 (0.94-1.23)	1.14 (0.96-1.36)
Sex				
Female	4452 (47.6)	268/4452 (6.0)	1 [Reference]	1 [Reference]
Male	4893 (52.4)	188/4893 (3.8)	1.25 (1.03-1.52)	1.10 (0.81-1.48)
Sexual orientation				
Heterosexual	8303 (88.8)	230/8303 (2.8)	1 [Reference]	NA
Maybe gay or bisexual	327 (3.5)	68/327 (20.8)	8.08 (6.23-10.46)	NA
Gay or bisexual	366 (3.9)	137/366 (37.4)	13.97 (11.32-17.23)	NA
Do not understand the question	262 (2.8)	12/262 (4.6)	1.81 (1.02-3.21)	NA
Refuse to answer	87 (0.9)	9/87 (10.3)	4.00 (2.11-7.58)	NA
Race and ethnicity				
Asian	557 (6.0)	23/557 (4.1)	0.88 (0.60-1.30)	0.79 (0.48-1.28)
Black	1641 (17.6)	101/1641 (6.2)	1.02 (0.79-1.31)	0.63 (0.46-0.89)
Latino or Hispanic	1545 (16.5)	68/1545 (4.4)	0.80 (0.58-1.10)	0.39 (0.24-0.63)
Native American	316 (3.4)	18/316 (5.7)	0.81 (0.51-1.29)	0.41 (0.21-0.81)
White	5207 (55.7)	242/5207 (4.6)	1 [Reference]	1 [Reference]
Other^c^	79 (0.8)	4/79 (5.1)	0.84 (0.32-2.20)	0.35 (0.08-1.60)
Religiosity				
Religious	6816 (72.9)	308/6816 (4.5)	0.94 (0.79-1.13)	1.08 (0.86-1.38)
Not religious	2529 (27.1)	148/2529 (5.9)	1 [Reference]	1 [Reference]
Household income, $				
≤24 999	1041 (11.1)	55/1041 (5.3)	1.44 (0.94-2.21)	1.50 (0.83-2.71)
25 000-49 999	1209 (12.9)	101/1209 (8.4)	2.02 (1.42-2.90)	2.06 (1.28-3.30)
50 000-74 999	1245 (13.3)	69/1245 (5.5)	1.65 (1.14-2.38)	1.81 (1.09-3.00)
75 000-99 999	1304 (14.0)	60/1304 (4.6)	1.37 (0.95-1.98)	1.33 (0.79-2.24)
100 000-199 999	3193 (34.2)	127/3193 (4.0)	1.23 (0.89-1.71)	1.50 (0.96-2.33)
≥200 000	1353 (14.5)	44/1353 (3.3)	1 [Reference]	1 [Reference]
Highest parental education				
High school education or less	879 (9.4)	59/879 (6.7)	1.45 (1.09-1.93)	1.67 (1.12-2.50)
College education or more	8466 (90.6)	397/8466 (4.7)	1 [Reference]	1 [Reference]

Abbreviations: aPR, adjusted prevalence ratio from Poisson regression model; NA, not applicable.

^a^ The model for the total sample represents the abbreviated output from the Poisson regression model with adjustment for age, sex, sexual orientation, race and ethnicity, religiosity, household income, parental education, and study site. The model for gay or bisexual and maybe gay or bisexual adjusts for age, sex, race and ethnicity, religiosity, household income, parental education, and study site.

^b^ The number of participants experiencing sexual orientation discrimination by each sociodemographic factor category divided by the total number of participants per sociodemographic variable.

^c^ This subcategory was listed as “other” but with no specific racial and ethnic groups defined, although write-ins were allowed. Race and ethnicity were reported by the parent and/or caregiver of the adolescent.

## Discussion

This cross-sectional analysis of early adolescents suggested that sexual minority identity, male sex, lower household income, and lower parental education were associated with higher sexual orientation discrimination. Interestingly, even adolescents who were questioning, refused to answer, or did not understand the sexual orientation question reported higher levels of sexual orientation discrimination than their heterosexual peers. It is possible that some of these early adolescents are not yet “out” given the age range of 10 to 13 years. Limitations of this study include the use of self-reported data. Structural factors may further influence sexual minority adolescents’ susceptibility to discrimination because those with lower household income or less educated parents, for example, may face compounded forms of bias. Studies have shown that sexual minority adolescents experiencing discrimination are more likely to experience mental health burdens^6^; therefore, these findings underscore the need to promote acceptance of diverse sexual orientations beginning in childhood to address sexual orientation discrimination among early adolescents.

## Notes

Supplement 1.
eMethods.

eReference.

Supplement 2.
Data Sharing Statement
