# Supplementary material for: Sexual Orientation Discrimination in Early Adolescents
Source: JAMA Netw Open. 2024 Oct 7;7(10):e2437985. doi: 10.1001/jamanetworkopen.2024.37985 (PMC11581521; doi:10.1001/jamanetworkopen.2024.37985)
Supplement: Supplement 1. — eMethods. eReference. [file jamanetwopen-e2437985-s001.pdf]

Supplemental Online Content

Nagata JM, Wong JH, Helmer CK, Domingue SK, Shim JE, Al-Shoaibi A. Sexual orientation discrimination in early adolescents. *JAMA Netw Open*. 2024;7(10):e2437985. doi: 10.1001/jamanetworkopen.2024.37985

eMethods.

eReference.

This supplemental material has been provided by the authors to give readers additional information about their work.

© 2024 Nagata JM et al. *JAMA Network Open.*

eMethods.

Study Design

The Adolescent Brain Cognitive Development (ABCD) Study is the largest, most demographically diverse prospective cohort study on adolescent brain health in the United States (US). The baseline sample (2016-2018) included 11875 children recruited through random probability sampling from schools within 21 catchment areas around the US. Parents/caregivers provided written informed consent and children provided written assent.

Measures

Sexual orientation status

Sexual orientation status was measured by sexual identity. Participants were asked, “Are you gay or bisexual?” Responses included “yes,” “maybe,” “no,” “don’t understand the question,” and “refuse to answer.”

Sexual orientation discrimination

Experiences of sexual orientation discrimination is a type of discrimination measured by the Perceived Discrimination Scale.[1] This scale measures adolescents’ perception of being treated unfairly due to sociodemographic characteristics. All participants were asked, “In the past 12 months, have you felt discriminated against because someone thought you were gay, lesbian, or bisexual?” Responses included “yes” and “no.”

Religiosity

Parents were asked, “What is the child’s religious preference?” Responses were combined into two categories: religious (Mainline Protestant, Evangelical Protestant, Historically Black Church, Roman Catholic, Jewish, Mormon, Jehovah’s Witness, Muslim, Buddhist, Hindu, Orthodox Christian, Unitarian, Other Christian) and not religious (Atheist, Agnostic, something else, nothing in particular).

Race and ethnicity

For ‘other’ race and ethnicity, no specific racial and ethnic groups were defined.

eReference.

1. Garnett BR, Masyn KE, Austin SB, Miller M, Williams DR, Viswanath K. The intersectionality of discrimination attributes and bullying among youth: an applied latent class analysis. J Youth Adolesc. 2014;43(8):1225-1239. doi:10.1007/s10964-013-0073-8

© 2024 Nagata JM et al. *JAMA Network Open.*
