# Supplementary material for: Sexual Orientation Discrimination in Early Adolescents
Source: JAMA Netw Open. 2024 Oct 7;7(10):e2437985. doi: 10.1001/jamanetworkopen.2024.37985 (PMC11581521; doi:10.1001/jamanetworkopen.2024.37985)
Supplement: Supplement 2. — Data Sharing Statement [file jamanetwopen-e2437985-s002.pdf]

Data Sharing Statement

Nagata. Sexual Orientation Discrimination in Early Adolescents. *JAMA Netw Open*. Published October 07, 2024. doi:10.1001/jamanetworkopen.2024.37985

Data
Data available: Yes
Data types: Deidentified participant data
How to access data: Data used in the preparation of this article were obtained from the ABCD Study (https://abcdstudy.org), held in the NIMH Data Archive (NDA).
When available: With publication

Supporting Documents
Document types: None

Additional Information
Who can access the data: Researchers whose proposed use of the data has been approved
Types of analyses: For a specified purpose
Mechanisms of data availability: With a signed data access agreement
